# A pilot study to assess the utility and perceived effectiveness of a tool for diagnosing feeding difficulties in children

**DOI:** 10.1186/s12930-015-0024-5

**Published:** 2015-07-31

**Authors:** Pankaj Garg, Jennifer A Williams, Vinita Satyavrat

**Affiliations:** Sir Ganga Ram Hospital, Rajinder Nagar, New Delhi, India; Abbott Nutrition Research and Development, Abbott Laboratories, Columbus, OH USA; Scientific and Medical Affairs, Abbott Nutrition International India, Mumbai, India

**Keywords:** Child, Feeding and eating disorders of childhood, Tool use behavior

## Abstract

**Background:**

Food dislikes in children may result in avoiding particular food/s with major sources of essential nutrients leading to increased risk of impaired growth or cognitive development and compromised immune function. It is necessary to identify conditions contributing to feeding difficulty and associated complications. An instrument was designed to assist diagnosis and management of children with feeding difficulties. The study was conducted to test utility of the “Identification and Management of Feeding Difficulties (IMFeD)” tool in Indian children.

**Methods:**

A prospective, cross-sectional study was conducted in Indian children between 2 and 10 years identified to have picky eating behaviour. After completion of both pro forma sections (parent and physician) of the IMFeD tool, the child’s specific feeding difficulty was diagnosed and appropriate nutritional and/or behavioural counselling was provided. The subjects were followed at 30 and 60 days post-intervention.

**Results:**

According to 66% of paediatricians the IMFeD tool was very easy to use. Approximately 85% of paediatricians required ≤20 min to administer the tool, diagnose the feeding difficulty(ies) and provide specific counselling or behavioural management. More than 70% of parents were satisfied and willing to accept the use of the IMFeD tool. After 60 days, 65% of the parents were either less worried or not worried at all about the feeding behaviour of the child using recommendations made on the basis of the IMFeD tool. The toolkit helped parents to know what to do if their child had a feeding problem. A total of 90% of the parents expressed that the tool is useful for assessing feeding difficulties in children.

**Conclusion:**

The IMFeD tool can be effectively used to identify feeding difficulties in Indian children. This toolkit also helps to offer nutritional and behavioural guidance as a part of the management.

## Background

Dietary intake of infants begins with a liquid diet, involves a transition to complementary foods by 6 months, and, by 24 months, most children primarily consume solid foods. The ages for typical progressions in feeding can vary and are influenced by, amongst other factors, maternal characteristics, ethnicity, and cultural traditions [1]. As consumption of food types and quantities changes, and infants and toddlers grow, children indicate their likes and dislikes for specific foods both behaviourally and verbally [2]. Their food dislikes may result in the avoidance of particular foods or groups of food that are major sources of essential nutrients and contribute to dietary variety. Children avoiding certain types of food/s may be perceived as picky eaters, problem feeders, or neophobics [1]. It is not uncommon for parents to approach family physicians and paediatricians with concerns about feeding problems in their child. The prevalence of picky eating behaviour in children ranges between 12 and 50% [3–7]. In one study surveying parental perceptions of children’s eating almost half of the primary caregivers noted that children are ‘all the time’ or ‘sometimes’ picky eaters [8].

Children with feeding difficulties are less likely to consume a nutritious diet than non-picky eaters [6, 9] and they are at risk for impaired growth [9, 10] or cognitive development [11] along with compromised immune function [12]. Parents often resort to different strategies such as using pressure or force with the child, in an effort to improve feeding behaviours [5]. However, the possible negative consequences that may result from attempts to change feeding behaviours, especially coercion, can compromise parent–child interactions [6, 7]. Early childhood feeding conflicts and struggles with food have been highlighted as risk factors for the later development of eating disorders such as bulimia or anorexia nervosa [13, 14].

To help determine appropriate intervention for children with feeding difficulties—whether it is reassurance, counselling to resolve behavioural problems (both the child and the feeder), nutritional intervention, or medical treatment—it is necessary to identify the specific conditions that contribute to a given feeding difficulty and its associated complications. The task of categorizing and treating children with feeding difficulties is often daunting for the paediatrician or family physician due to time constraints and a lack of expertise in this particular field.

**Fig. 1:**
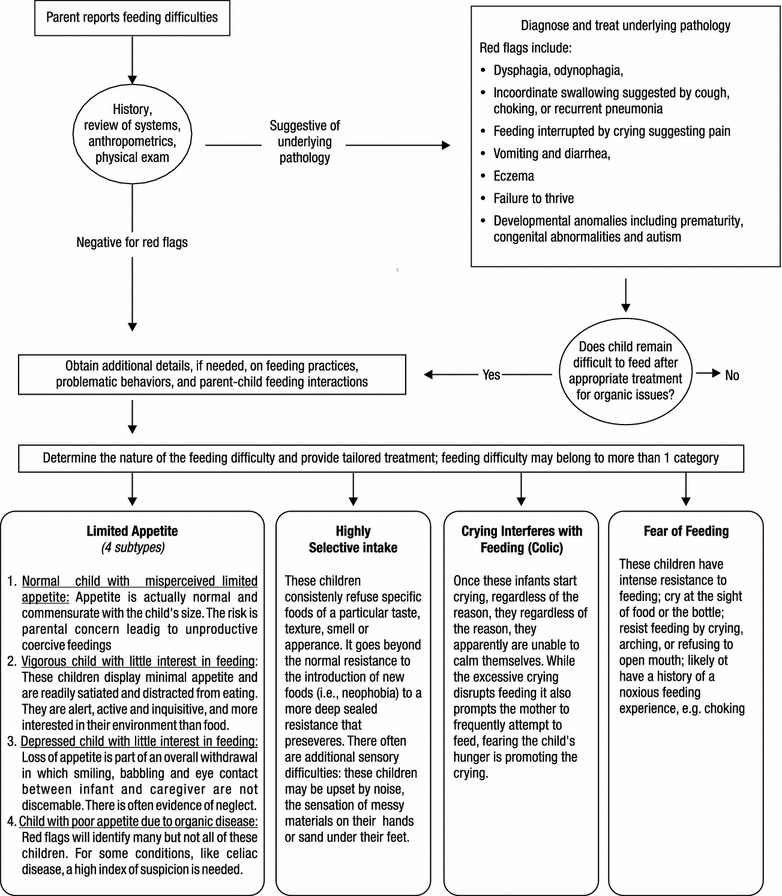
Diagnosis of common types of feeding difficulties in young children [3, 4].

**Fig. 2:**
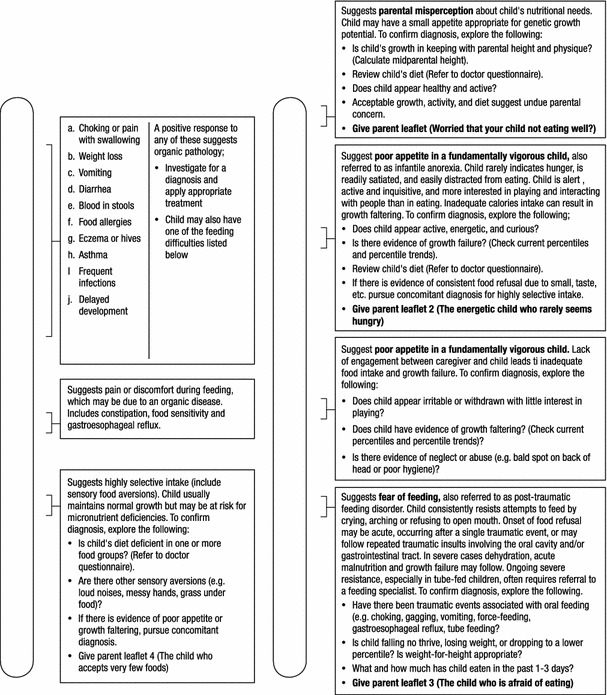
IMFeD tool: diagnostic framework [4].

**Fig. 3:**
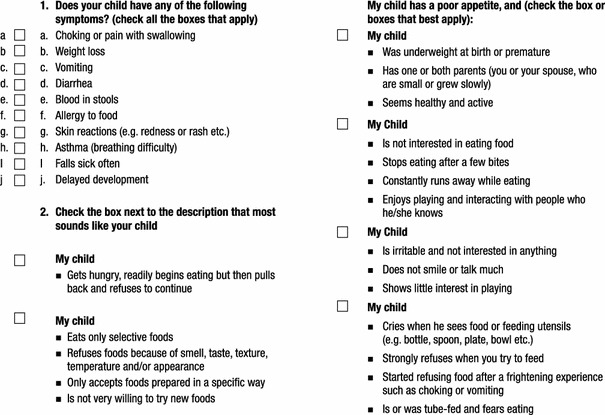
IMFeD tool: parent questionnaire.

**Fig. 4:**
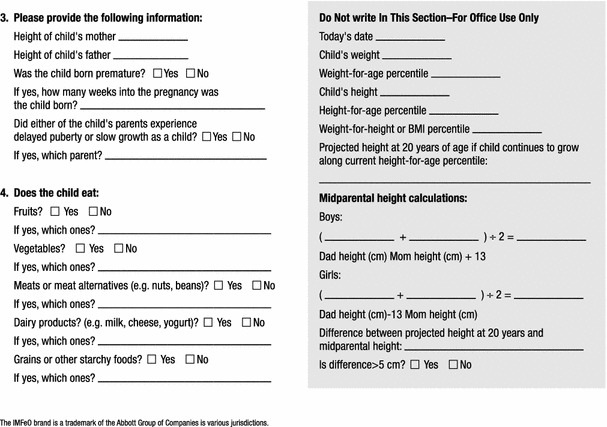
IMFeD tool: physician questionnaire.

To overcome the limitations detailed above, the Identification and Management of Feeding Difficulties for Children (IMFeD) tool was developed based on the research by Chatoor [4] for the classification of feeding difficulties, and further complemented by information derived from the experiences of Kerzner [3], who has helped provide a structured approach for managing a child with a feeding difficulty (Fig. 1). The IMFeD tool consists of the diagnostic framework based on six distinct types of feeding difficulty categories, presented in Fig. 2, a parent questionnaire (Fig. 3), and a physician questionnaire (Fig. 4).

A two-part questionnaire and pro-forma developed as part of a ‘tool kit’ was designed to be utilized globally to assist a physician in diagnosing and managing children with feeding difficulties. The tool is currently being validated against professional feeding difficulties assessments [3]. Once a diagnosis or set of diagnoses is established by the physician using the pro-forma with the parent or caregiver to support the clinical reasoning process, a structured and specific set of guidelines can be provided for treatment of the specific feeding difficulty(ies) for an individual child.

### Objective

The tool is currently being validated and the purpose of the present study is to test the usefulness of the draft version of the tool as part of the developmental process. The two primary goals for the current study were to determine how the tool would be used in India specifically, and what physicians and caregivers thought about the tool.

## Methods

This was a prospective, cross-sectional study conducted at 10 distinct study sites across India. The study was conducted after receiving approval from an Independent Ethics Committee. The children aged 2–10 years whose parent/caregiver complained that their child had two of mentioned picky eating habits like (1) the child is too selective or ‘picky’; (2) the child eats too little; (3) the child fails to advance to more complex foods; or (4) the child only eats ‘junk food’ were included in the study. Overweight, obese or children at risk being for overweight, or suffering from chronic medical conditions, or having a chronic mental or developmental problem were excluded from the study. Baseline and demographic information of the subjects was collected after their enrolment in the study.

The IMFeD tool helps paediatricians identify common feeding difficulties in children and also offers some approaches for managing them by providing suggestions for parent education. Although feeding difficulties are commonly recognized problems, the types of feeding problems found in a specific population or country have not been studied broadly.

The paediatrician administered the first component of the IMFeD tool with the parent/caregiver of the child. As the questionnaire was in English, only parent/caregivers who understood English were enrolled in the study. After completion of both sections of the IMFeD tool (parent and physician), the child’s specific feeding difficulty(ies) was diagnosed by the paediatrician. Based on the type(s) of diagnosed feeding difficulty, appropriate nutritional and/or behavioural counselling was provided to the parent/caregiver. All enrolled participants were followed up at 30 and 60 days post-intervention, and changes in feeding behaviours, if applicable, were captured during these follow-up visits. Additionally, at the exit visit, a questionnaire was administered to both the paediatrician and parent to determine the acceptance of the IMFeD tool.

### Statistics

This study is based on a convenience sample of children identified by their parents as having feeding difficulties. We planned to enrol approximately 400 children. Statistical analysis was performed using the SPSS software package version 10.0 (Softonic^**®**^). Demographic and baseline data (n, mean, standard deviation, range) were calculated for continuous variables, while counts and percentages were calculated for categorical variables. Variables such as the acceptance of the IMFeD tool by the physician and the parent/caregiver were estimated and presented with frequency counts and percentages. All values were reported based on two-sided distribution, and all statistical tests were interpreted at a 5% level of significance.

## Results

The study was conducted between March 2011 and March 2012. Against the estimated plan of 400 children enrolment, 383 children across ten centres in India were enrolled. Three children were lost to follow-up, hence the final evaluable set of subjects was 380 children. Demographic data for subjects are presented in Table 1.

**Table 1:** Demographic and baseline characteristics

	Boys	Girls	P value
Sample size	222	158	
Age in yrs, mean ± SD	4.59 ± 02.03	4.61 ± 02.07	0.9254
Weight in kg, mean ± SD	15.49 ± 04.62	15.18 ± 04.62	0.5195
Height in cm, mean ± SD	101.15 ± 15.00	99.41 ± 16.35	0.2907

The results showed that the “IMFeD” tool helped paediatricians identify the “type of feeding difficulty” among those children considered to be picky eaters. Table 2 shows the prevalence of the individual diagnosis categories of “feeding difficulty” based on the IMFeD tool, with the most common feeding difficulty assessed as “poor appetite in fundamentally vigorous child” followed by “highly selective intake”. A total of 124 children (33%) presented with more than one feeding difficulty (Table 2).

**Table 2:** Diagnosis of feeding difficulties based on the IMFeD tool

Feeding difficulty category	No. of cases (N = 380)	Percentage (%)
Organic disease	1	0.3
Highly selective intake	150	39.5
Parental misperception	97	25.5
Fundamentally vigorous child	231	60.8
Apathetic & withdrawn child	7	1.8
Fear of feeding	18	4.7

**Fig. 5:**
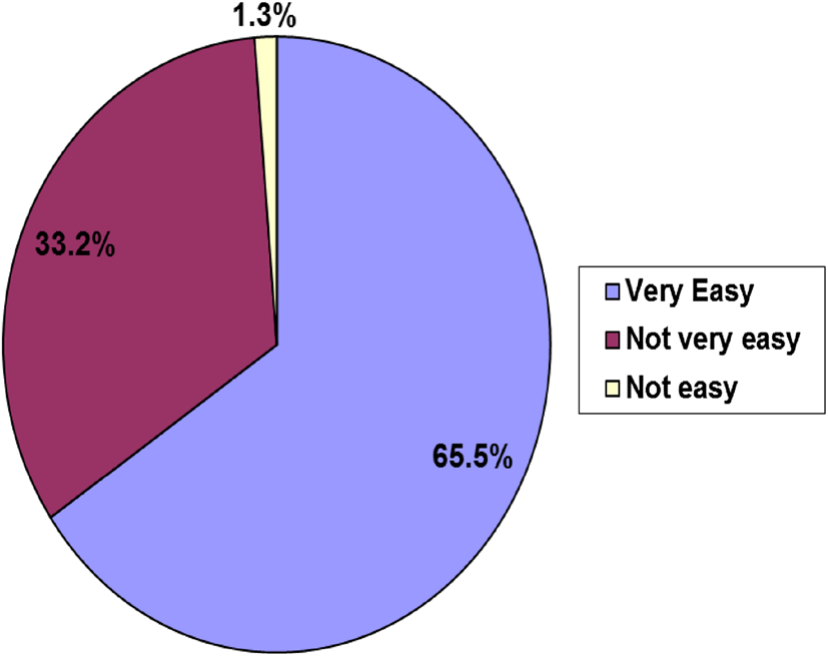
Physician opinion about application of the IMFeD tool.

**Fig. 6:**
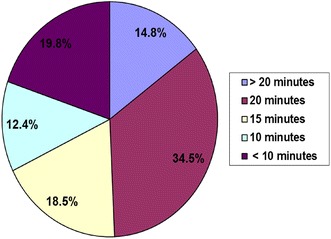
Time required by physician to administer the IMFeD tool.

The study also assessed the acceptance of the IMFeD tool by study paediatricians as a process aid for the diagnosis of paediatric feeding difficulties. According to 66% of the paediatricians, the IMFeD tool was very easy to use (Fig. 5), and approximately 85% of the study paediatricians (Fig. 6) required 20 min or less to administer the tool and diagnose the feeding difficulty(ies), along with conducting specific counselling or behavioural management.

**Fig. 7:**
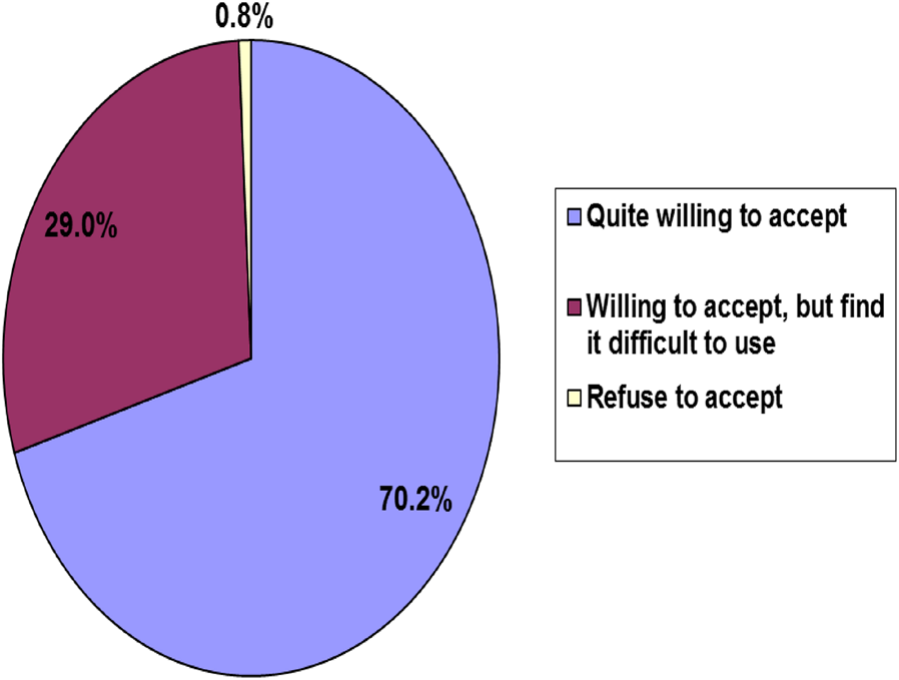
Parent attitudes regarding use of the IMFeD tool.

**Fig. 8:**
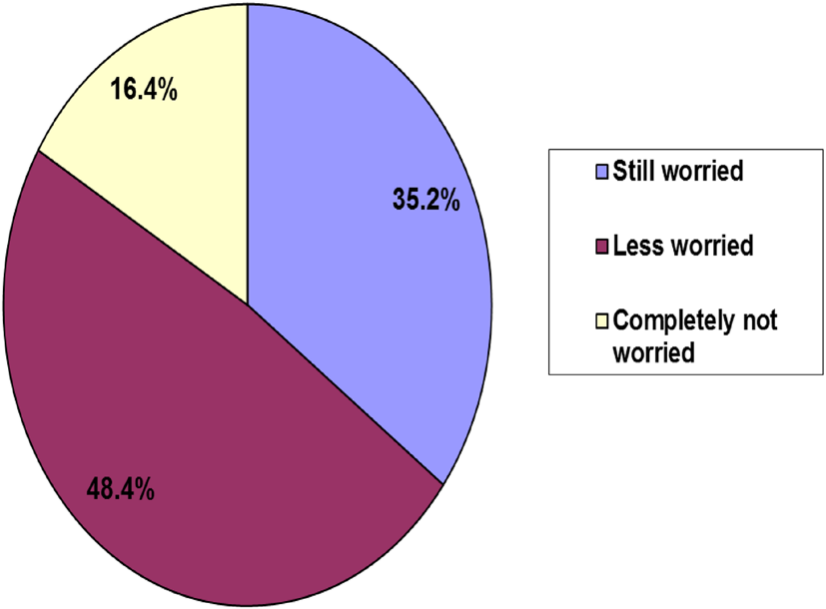
Parent attitudes about child’s feeding difficulty after use of the IMFeD tool.

More than 70% of parents were satisfied and willing to accept the use of the IMFeD tool to manage their child’s feeding issues (Fig. 7). At the end of the 60 day study period utilizing the recommendations from the IMFeD tool, 65% of the parents were either less worried or not worried at all about the feeding behaviour of their child (Fig. 8). On completion of the study, 51% parents were confident and believed that they knew what to do if their child had a feeding problem and were also able to apply the recommended strategies to improve their child’s appetite and feeding behaviour. Sixty-two percent of the study parents felt that the IMFeD tool used by the paediatrician for their child’s eating problems was effective, and 90% of the parents expressed that the IMFeD tool is a good instrument to assess the feeding difficulties in children.

## Discussion

The continuum of feeding difficulties can range from mild behavioural issues to major organic disorders. Feeding problems such as organic disease, infantile anorexia, food allergies, food aversion, food selectivity, food refusal, selective eating, colic, fear of feeding, post-traumatic feeding disorder, and even parental misperception all fall somewhere on this scale of severity. Interestingly, organic disease as a cause is implicated in only 5% of feeding difficulty diagnoses [15]. If not treated, feeding difficulties may cause adverse implications such as nutritional deficiencies, failure to thrive, or chronic feeding aversion. A large scale, longitudinal study of young children in Quebec found picky eaters were twice as likely as non-picky eaters to be underweight at 4.5 years of age [7]. A long-term follow-up study of Norwegian children with early refusal to eat demonstrated that picky and problematic eating behaviours can persist up to 9 years of age [16]. Implications can extend beyond growth impairment to emotional and cognitive issues.

Paediatricians are instrumental for resolving feeding issues, and they commonly address these conditions in the clinical setting. However, paediatricians, with busy schedules and no standard protocol available for the treatment of feeding difficulties, may not be able to provide specific counselling to their paediatric patients and their parents/caregivers.

Some studies describe picky or fussy eating in terms of a limited variety of food in the diet [8]. This study showed that the most common feeding difficulty for this specific study population suggested by the physicians using the IMFeD tool were ‘fundamentally vigorous child’ followed closely by ‘highly selective intake’ and ‘parent misperception’. The prevalence of specific feeding difficulties may differ from population to population, country to country, and within different age groups studied. Because paediatricians are often the key stakeholders in the management of feeding difficulties, it was important to assess their opinion on the ease of use and average time spent while diagnosing feeding difficulty(ies) in a child with the IMFeD tool. More than 90% of paediatricians said that the tool was easy or very easy to use, and the time taken for diagnosis was typically less than 20 min. Approximately 20% of the study paediatricians were able to implement the tool in less than 10 min. This implies that with continuous and regular use, the paediatricians became more acquainted with the tool, which reduced the time required for its implementation. Continued familiarity and experience with the IMFeD tool would facilitate ready adoption into routine practice by the paediatrician, possibly helping to alleviate anxiety or errors during the diagnosis of feeding difficulties. Parent participation is also very important during implementation of the management strategies designed for the diagnosed feeding difficulty. For intervention success it is important for the parent to accept the utility of the IMFeD tool and follow the counselling provided. In this study, the majority of parents willingly accepted the use of the IMFeD tool by the paediatrician.

Previous research has shown that picky eating can cause considerable parental concern over the child’s physical and mental health [11]. Notably, more than half of the parents in this study experienced a significant decrease in their concern about their child’s feeding problem after use of the IMFeD tool.

This study has some limitations. Although the tool is not yet validated, it is currently being validated against standardized feeding difficulties assessments [3]. Secondly, convenience sampling used in this study is associated with many limitations. As the sampling was not randomized, the findings of the study may not be generalized more broadly to the entire population. A larger study with randomized sampling is required to determine whether our study results would hold true for a more representative sample of the population. In this study, only opinions of the physician are reported while health outcome measures are not evaluated. Further studies with data at minimum two follow up points are recommended to evaluate the health outcomes in children.

## Conclusion

This pilot study shows that the IMFeD tool can be applied by paediatricians in their routine clinical practice to identify the feeding difficulties in Indian children. The IMFeD tool helps in diagnosing the type of feeding difficulty, and also offers nutritional and behavioural guidance as a part of the management and improvement of feeding difficulties. However, larger comparative studies need to be conducted to prove that the IMFeD tool is a useful instrument for diagnosing feeding difficulties and also enhancing nutritional status in Indian children.

## Abbreviation

IMFeD: Identification and Management of Feeding Difficulties
